# Discontinuation of Tyrosine Kinase Inhibitors in Chronic Myeloid Leukemia With Losing Major Molecular Response as a Definition for Molecular Relapse: A Systematic Review and Meta-Analysis

**DOI:** 10.3389/fonc.2019.00372

**Published:** 2019-05-14

**Authors:** Kang-kang Chen, Tai-feng Du, Pei-sheng Xiong, Guan-hua Fan, Wei Yang

**Affiliations:** ^1^Department of Preventive Medicine, Shantou University Medical College, Shantou, China; ^2^Department of Thoracic Surgery, Administrative Office, Shantou University Medical College Cancer Hospital, Shantou, China

**Keywords:** tyrosine kinase inhibitors, chronic myeloid leukemia, treatment-free remission, treatment discontinuation, deep molecular response

## Abstract

**Background:** A new goal in treatment of chronic myeloid leukemia (CML) in patients with stable deep molecular response (DMR) is maintaining durable treatment-free remission (TFR) after discontinuing tyrosine kinase inhibitor (TKI) treatment.

**Methods:** We conducted a systematic review and meta-analysis focusing on the efficacy and safety of TKI discontinuation but also exploring the factors contributing to successful TFR.

**Results:** The search yielded 10 trials including 1,601 patients. For patients who discontinued TKIs, the estimated weighted mean incidence of major molecular relapse was 16% (95%CI: 11–21), 34% (95%CI: 29–38), 39% (95%CI: 35–43) and 41% (95%CI: 36–47) at 3, 6, 12, and 24 months, respectively. Of these, 39, 82, and 95% of molecular losses occurred within the first 3, 6, and 12 months. In safety analysis, among patients without TFR, 98% (95% CI: 96–100) were sensitive to TKI retreatment. No new safety issues were identified except TKI withdrawal syndrome, which appeared during the early TFR phase, with a weighted mean incidence of 27% (95%CI: 19–35). Our subgroup analysis suggested better TFR associated with interferon therapy (*P* = 0.007), depth of molecular response (*P* = 0.018) and duration of DMR (*P* < 0.001).

**Conclusions:** TFR as an extension of an approach to optimize management of CML is clinically feasible in approximately 59% of patients with sufficient TKI response. In the remaining 41% of patients with molecular relapse, discontinuing TKIs had no negative impact on clinical outcomes. Given the high heterogeneity among studies, the role of these predictors for successful TFR still requires further investigation.

## Introduction

The prognosis of patients with chronic myeloid leukemia (CML) has significantly improved over the past 2 decades since the 5 small-molecule tyrosine kinase inhibitors (TKIs; imatinib, danstinib, nilotinib, bosutinib, and ponatinib) were applied to CML therapy (1). The life expectancy of CML patients who may once have died within 7 years of diagnosis in the pre-TKI era is now more likely close to that of the general population (2, 3).

However, previous studies demonstrating that TKI monotherapies are unable to completely eliminate Philadelphia chromosome-positive hematopoietic stem cells led to the recommendation that patients remain on TKI treatment indefinitely (1, 4). Lifelong treatment for all patients may place them at potential risk of treatment side effects or off-target effects, further compromising their health-related quality of life (5, 6). In addition, the high cost of out-of-pocket expenses for TKI treatment combined with the increasing number of CML patients due to effective TKI therapy implies a considerable financial burden for individuals and communities (3, 7). Such disadvantages of indefinite TKI treatment leads to exploring the feasibility of discontinuing drug therapy among patients with sufficient TKI response.

Convincing results for treatment-free remission (TFR) were originally derived from the prospective, multicenter study STIM, in which the criterion for imatinib discontinuation was a minimum of 3 years of imatinib treatment and > 2 years of sustained undetectable BCR-ABL1 transcripts (8). Approximately, 40% of patients in that study remained in TFR for > 3 years. Since then, many studies have evaluated the potential for TFR after first- or second-line TKI treatment: all confirmed the high possibility of TFR in patients with stable deep molecular response (DMR) for several years.

As knowledge from TFR studies continues to accumulate, the National Comprehensive Cancer Network (NCCN) has recently incorporated this treatment strategy into its guidelines (9). Currently, TFR as an extension of an approach to optimize management of CML is about to officially shift from clinical trials to routine clinical practice. However, several questions with no clear answers remain. First, various TKI discontinuation studies have shown discordance in the incidence of molecular relapse, ranging from ~30 to 60%. Second, the time distribution of adverse events (AEs) as well as TKI withdrawal syndrome (TKI-WS; consisting mainly of musculoskeletal pain and pruritus, etc.) in TFR have not been clearly elucidated. These discordances may induce confusion about clinical decisions in patients or even physicians and significantly affect compliance with drug withdrawal strategies.

In addition, if the patient selection criteria cannot be found, all CML patients have to undergo frequent monitoring after TKI discontinuation, which imposes a huge financial burden on patients and also causes a serious waste of medical resources. An active field of current research is identifying the potential predictors of successful or feasible TFR attempts for a given patient. Various prognostic factors have been discovered, but the findings from most studies are contradictory, so clear-cut conditions that favor TKI discontinuation are difficult to define. In the STIM 1 (8), EURO-SKI (10), and ENESTfreedom (11) studies, the probability of TFR was greater with low than high Sokal scores, whereas the TWISTER (12), JALSG-STIM213 (13), and KID (14) studies found no correlation between Sokal score and TFR rates. In addition to patient characteristics, the ENESTfreedom study (15) showed that the depth of molecular response was a better predictor of TFR, but the EURO-SKI study (10) found that high probability of remaining TFR was associated with duration of DMR before TKI discontinuation and total duration of TKI therapy. Similarly, data from the DADI (16) and STOP 2G-TKI (17) studies suggested that probability of TFR was lower for patients who switched to new-generation TKIs because of imatinib resistance or suboptimal response than those who switched because of prior imatinib intolerance; in contrast, Mahon et al. (18) found that prior imatinib resistance or intolerance did not affect TFR rates.

Given the inconsistency of the above findings, together with recently published studies to add to the evidence base of TFR, we performed a meta-analysis of TKI discontinuation. The primary objectives were to evaluate the efficacy and safety of TKI discontinuation. The secondary objective was to explore factors contributing to successful TFR.

## Methods

This meta-analysis was performed according to the Preferred Reporting Items for Systematic Reviews and Meta-Analyses (PRISMA) statement (19).

### Search for Articles

To identify published studies reporting TFR rates in patients with sufficient TKI response, we searched PubMed, EMBASE, and Cochrane Central Register of Controlled Trials databases from inception to October 25, 2018, by using the terms “Leukemia, Myelogenous, Chronic, BCR-ABL Positive,” “tyrosine kinase inhibitor,” “imatinib,” “dasatinib,” “nilotinib,” “bosutinib,” “ponatinib,” “stopping,” “discontinuation,” and “treatment free remission.” We also searched reference lists of included reports for any relevant studies. No restrictions were applied for language and publication status.

### Eligibility Criteria and Study Selection

We included studies with the following inclusion criteria: (1) enrolling CML patients who were Philadelphia chromosome–positive who received TKIs and reached DMR before entering the TFR phase; (2) adopting loss of major molecular response (MMR) as a definition for molecular recurrence; (3) reporting at least one TFR rate during follow-up or providing a corresponding Kaplan-Meier curve; and (4) enrolling adult patients. Exclusion criteria were (1) enrolling participants who never received TKIs; (2) enrolling participants who had undergone allogeneic hematopoietic stem-cell transplantation or receiving treatment for other malignant disorders; (3) participants continuing drug treatment (such as interferon alfa) during the TFR phase; and (4) case reports or abstracts.

Two researchers (Chen and Du) independently screened abstracts and titles, then read the full text of relevant studies. Any disagreements were resolved by consensus or by a third reviewer (Fan).

### Data Extraction and Risk of Bias Assessments

Two researchers (Chen and Du) independently extracted information on lead author; publication year; participant characteristics (including age, sex, Sokal risk score and interferon treatment); type of first-line TKI therapy; treatment history; sample size; number of patients with relapse at 3, 6, 12, and 24 months; total duration of TKI therapy; duration of DMR before TKI discontinuation; depth of molecular response before TKI discontinuation; TKI-WS; and risk of bias elements. One researcher extracted data from included studies, and another confirmed the accuracy of the data. Any disagreements were resolved by consensus.

The risk of bias for the included observational studies was independently evaluated by 2 researchers (Chen and Du) according to the modified version of the Newcastle-Ottawa Scale (NOS). For each included study, the following nine components were used to assess bias: (1) study population clearly defined, (2) cohort representative for CML-CP patients with sufficient TKI response under TFR conditions, (3) definition of molecular response according to the international scale, (4) demonstration that the outcome of interest was not present at start of study, (5) consecutive patients included, (6) assessment of outcome, (7) follow-up long enough for outcome to occur (≥2 years), (8) adequacy of follow-up of cohorts, and (9) prospective study. The nine components are scored from 0 and 9, with two quality categories as follows: a study with >5 points was defined as having low risk of bias and ≤ 5 points as high risk of bias. Disagreements were determined by discussion or with a third reviewer (Fan).

### Statistical Analysis

We obtained the required data directly from the original article or based on the relevant Kaplan-Meier curve provided in the original article, combined with the Engauge Digitizer 4.1 and the excel file provided by Tierney et al. (20) From the abstracted data, the proportion of patients with molecular relapse and 95% confidence intervals (CIs) for each study were calculated, then the variance of individual studies was stabilized by Freeman-Tukey double arcsine transformation. A fixed effects model was used to pool similar outcomes unless we detected significant statistical heterogeneity (*I*^2^ > 50%), then a random effects model was chosen as the primary analytical model because of its conservative estimates. To evaluate statistical heterogeneity between summary data, we used the *I*^2^ statistic, which reflected the total variability across various studies explained by study populations, protocols, interventions or outcomes rather than chance or random error. *I*^2^ <25% reflected mild heterogeneity, 25–50% moderate heterogeneity, and >50% severe heterogeneity.

To identify the factors contributing to successful TFR, we performed exploratory sub-analyses according to participant characteristics (including age, sex ratio, and Sokal score), interferon therapy, type of first-line TKI therapy, treatment history, total duration of TKI therapy before TKI discontinuation, duration of DMR before TKI discontinuation, depth of molecular response before TKI discontinuation, and TKI-WS. Analysis was performed to evaluate whether the difference between the subgroups was statistical significance.

Because almost all studies included in this meta-analysis were observational, we did not assess potential publication bias because statistical non-significance is not likely to bias publications in the context of observational studies. Analyses were performed with STATA 14.0 (Stata Corp., College Station, TX). *P* < 0.05 was considered statistically significant.

## Results

### Studies Retrieved and Characteristics

**Figure 1:**
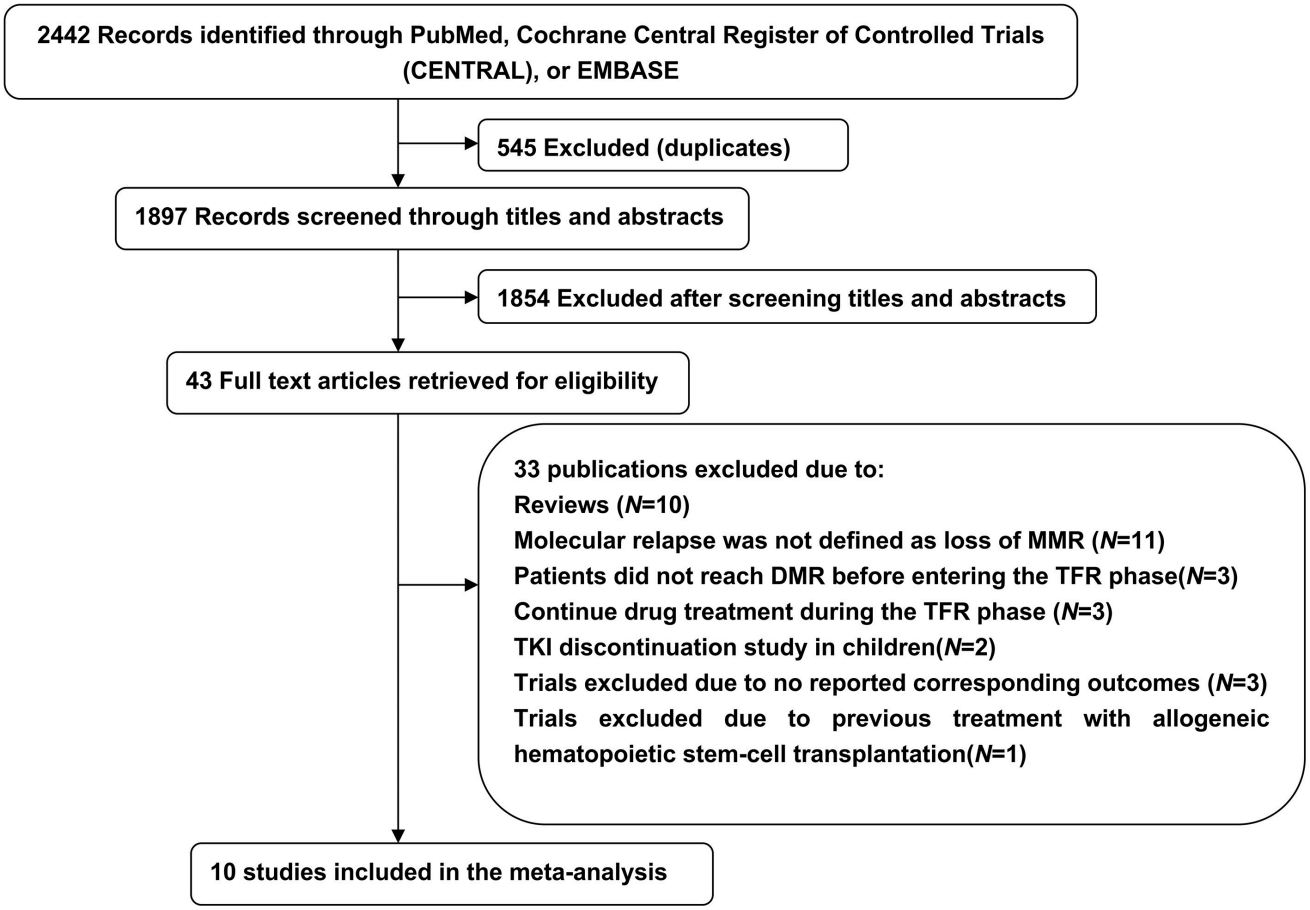
Literature search and screening process.

Our initial search yielded 2,442 potentially relevant studies; 545 were excluded because of duplicate publications and 1,854 were further excluded after screening titles and abstracts. The remaining 43 articles were analyzed and 33 were excluded: 10 were reviews, 20 were incompatible with our previously established eligibility criteria, and 3 did not report corresponding outcomes. Thus, during 2012–2018, 10 trials included 1,601 patients met the inclusion criteria and were summarized in this meta-analysis (Figure 1).

Six trials (10, 13, 14, 21–23) investigated the potential for TFR after first-line imatinib treatment, 1 (15) the potential after first-line nilotinib treatment, and 3 (17, 18, 24) the potential after second-line or subsequent second-generation TKI treatment. All included trials defined sustaining stable DMR for a significant time before entering the TFR phase and loss of MMR as a trigger for TKI re-treatment. Other detailed characteristics of the included trials are in Tables 1, 2.

**Table 1:** Participant characteristics and loss of major molecular response (MMR) rates.

**References**	**Sample size**	**Male ratio (%)**	**Age**	**Sokal (%)**			**No. of patients with loss of MMR (%)**			
				**Low**	**Intermediate**	**High**	**3 months**	**6 months**	**12 months**	**24 months**
Takahashi et al. (23)	43	44	57	25 (58.1)	15 (34.9)	3 (7)	4 (9.3)	11 (25.6)	14 (32.6)	17 (39.5)
Rousselot (22)	80	52	55	41 (51.3)	22 (27.5)	16 (20)	25 (31.3)	25 (31.3)	28 (35)	29 (36.3)
Mori et al. (21)	108	59	49	40 (37)	29 (26.9)	8 (7.4)	6 (5.6)	30 (27.8)	41 (38)	52 (48.1)
Lee et al. (14)	90	42	56	29 (32.2)	23 (25.6)	15 (16.7)	20 (22.2)	29 (32.2)	34 (37.8)	37 (41.1)
Ross et al. (15)	190	50	55	62 (32.6)	50 (26.3)	28 (14.7)	25 (13.2)	70 (36.8)	92 (48.4)	97 (51.0)
Rea et al. (17)	60	37	60	32 (53.3)	16 (17.8)	9 (15)	11 (18.3)	18 (30)	21 (35)	24 (40)
Takahashi (13)	68	62	55	51 (75)	6 (8.8)	11 (16.2)	9 (13.2)	19 (27.9)	22 (32.4)	24 (35.3)
Takahashi et al. (24)	78	58	57	44 (56.4)	17 (21.8)	16 (20.5)	NR	25 (32.1)	25 (32.1)	29 (37.2)
Saussele (10)	758	52	60	259 (34.2)	197 (26)	128 (16.9)	136 (17.9)	323 (42.6)	340 (44.9)	379 (50)
Mahon et al. (18)	126	44	56	NR	NR	NR	NR	NR	34 (26.9)	36 (28.5)

*NR, not reported*.

**Table 2:** Treatment characteristics for patients in the included trials.

**References**	**Interferon treatment (%)**	**Type of TKI therapy**	**Treatment history**	**Total duration of TKI therapy (months)**	**Duration of DMR before TKI discontinuation (months)**	**Depth of molecular response before TKI discontinuation**	**TKI-WS (%)**	**Risk of bias**
Takahashi et al. (23)	58	IM	1st line	45	27.4	CMR	NR	H
Rousselot (22)	52	IM	1st line	79	41	MR^5^	NR	L
Mori et al. (21)	33	IM	1st line	103	25.8	CMR	NR	L
Lee et al. (14)	9	IM	1st line	81	39.9	MR^5^	30	L
Ross et al. (15)	0	NIL	1st line	43	18.3	MR^4.5^	24.7	L
Rea et al. (17)	28	NIL/DAS	1st/2nd/3rd line	76	29	MR^4.5^	NR	L
Takahashi (13)	19	IM	1st line	97	66.9	MR^4.5/5^	14.7	L
Takahashi et al. (24)	15.4	NIL	2nd line	99	51.1	MR^4.5/5^	14.1	L
Saussele (10)	12	IM/NIL/DAS	1st/2nd line	90	NR	MR^4^	30.7	L
Mahon et al. (18)	NR	NIL	2nd line	88	32.8	MR^4.5^	48	L

*TKI, tyrosine kinase inhibitor; TKI-WS, tyrosine kinase inhibitor withdrawal syndrome; DMR, deep molecular response; NR, not reported; H, high risk of bias; L, low risk of bias; IM, imatinib; NIL, nilotinib; DAS, dasatinib; CMR, undetectable BCR-ABL1 by qPCR as determined by local laboratories*.

In addition to one study (23), the methodological quality of the included studies evaluated by the NOS was high (Table S1). On sensitivity analysis to detect potential bias due to the quality of the included studies, the bias was generally acceptable, with only 1 study (18) having a major impact on the success rate of TKI discontinuation (Figure S1).

### Molecular Relapse Rate and Safety (Figure 2)

The estimated weighted mean incidence of loss of MMR was 16% (95%CI: 11–21; *I*^2^ = 79%), 34% (95%CI: 29–38; *I*^2^ = 63%), 39% (95%CI: 35–43; *I*^2^ = 50%) and 41% (95%CI: 36–47; *I*^2^ = 75%) at 3, 6, 12, and 24 months, respectively, for patients who discontinued TKIs. Of these, 39, 82, and 95% of molecular losses occurred within the first 3, 6, and 12 months, respectively. We performed a sensitivity analysis of MMR at 3, 6, 12, and 24 months without the trial by Takahashi et al. (23), which had high-risk of bias on methodological quality: the weighted mean loss of MMR at each study endpoint was stable.

**Figure 2:**
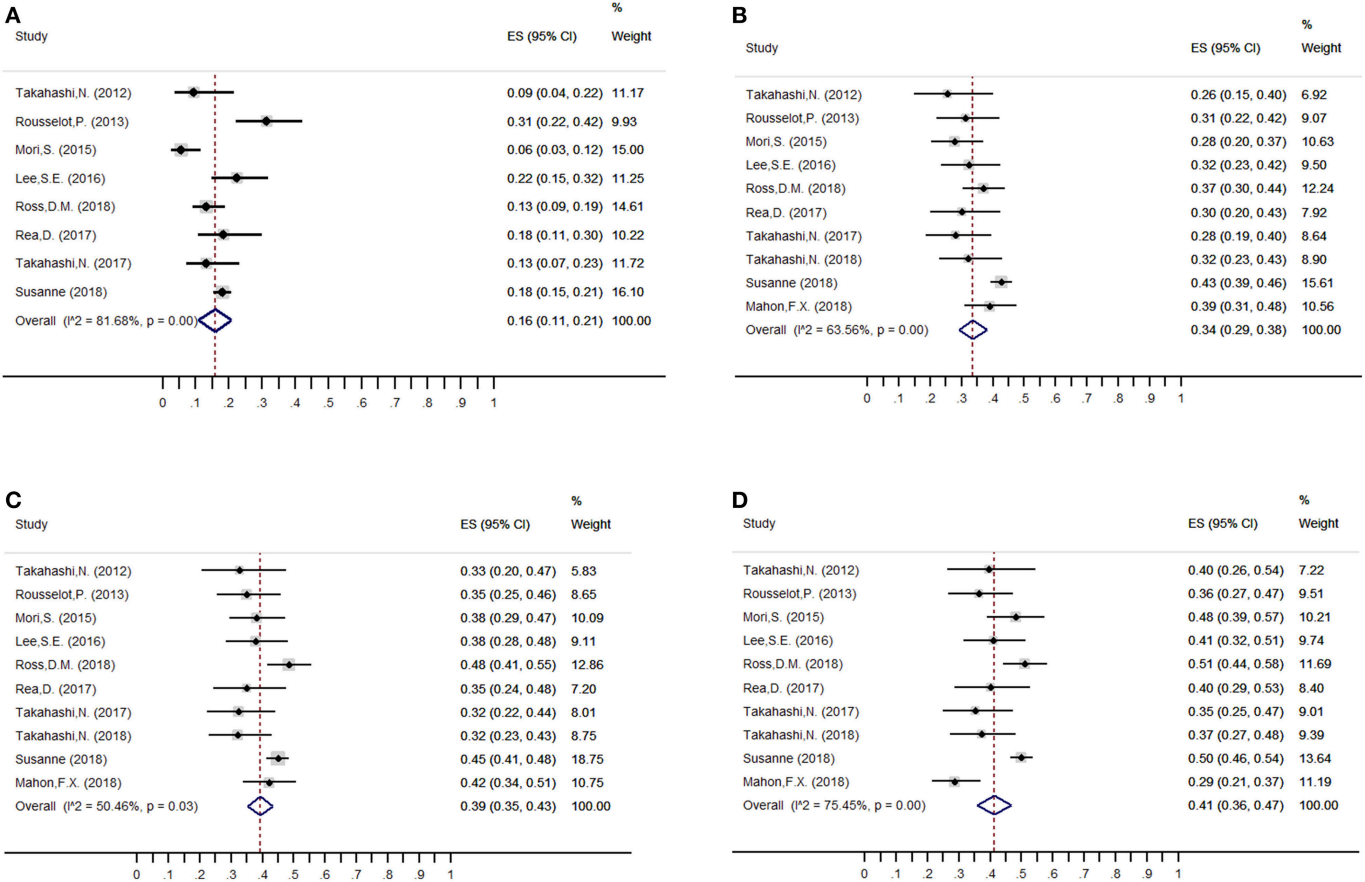
Overall weighted mean molecular relapse rate at 3, 6, 12, and 24 months after imatinib discontinuation. **(A)** 3 months; **(B)** 6 months; **(C)** 12 months; **(D)** 24 months.

Among the patients who restarted TKI treatment for molecular relapse, 98% (95%CI: 96–100; *I*^2^ = 0) regained MMR at the end of follow-up. Furthermore, no CML-related deaths were reported, and only one patient in the A-STIM study showed progression to blast crisis after imatinib resumption (0.01%, 95%CI: 0–0.07; *I*^2^ = 0). For AEs, no new safety issues were identified after TKI discontinuation, except TKI-WS, which appeared during the early TFR phase, with a weighted mean incidence of 27% (95%CI: 19–35; *I*^2^ = 88.98%).

### Subgroup Analyses

#### Interferon Therapy (Figure 3A, Table 3)

The median proportion of patients receiving interferon in each study was 19% (10, 13–15, 17, 21–24). The estimated loss of MMR was 48% (95%CI: 46–51) and 40% (95%CI: 35–46) in the nine studies that included less than 19% (10, 14, 15, 24) and at least 19% (13, 17, 21–23) of patients with interferon treatment.

**Figure 3:**
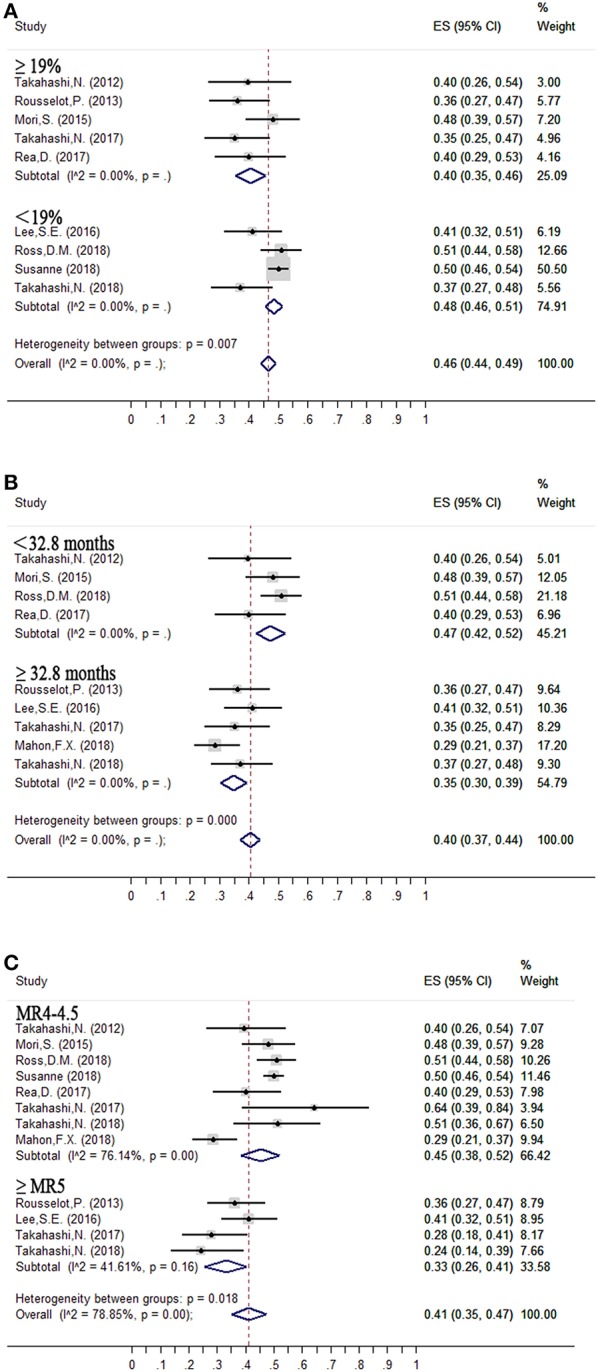
Subgroup analysis of factors contributing to successful treatment-free remission. **(A)** Interferon therapy; **(B)** Duration of DMR before tyrosine kinase inhibitor (TKI) discontinuation; **(C)** Depth of molecular response before TKI discontinuation.

**Table 3:** Subgroup analysis of predictors of successful TFR.

**Variable**	**No. of trials**	**No. of participants**		**Loss of MMR rate (95%CI)**	***P*-value**
		**No. of patients with relapse**	**Total**		
Age					0.526
<56	4	202	446	0.43 (0.35–0.51)	
≥56	6	522	1,155	0.40 (0.31–0.48)	
Male ratio					0.706
<51%	5	211	509	0.40 (0.31–0.49)	
≥51%	5	513	1,092	0.42 (0.35–0.50)	
Sokal score					0.204
<16.2%	4	190	401	0.47 (0.41–0.52)	
≥16.2%	5	498	1,074	0.44 (0.33–0.48)	
**Interferon therapy**					**0.007**
<19%	4	542	1,116	0.48 (0.46–0.51)	
≥19%	5	146	359	0.40 (0.35–0.46)	
Type of first-line TKI therapy					0.574
Imatinib	6	538	1,147	0.43 (0.37–0.49)	
Nilotinib	4	186	454	0.39 (0.28–0.50)	
Treatment history					0.586
First-line treatment	6	256	579	0.43 (0.37–0.49)	
Second-line treatment	4	468	1,022	0.39 (0.27–0.51)	
Total duration of TKI therapy before TKI discontinuation					0.668
<84.5	5	204	463	0.43 (0.36–0.49)	
≥84.5	5	520	1,138	0.40 (0.31–0.50)	
**Duration of DMR before TKI discontinuation**					**<0.001**
<32.8	4	190	401	0.47 (0.42–0.52)	
≥32.8	5	155	422	0.35 (0.30–0.39)	
**Depth of molecular response before TKI discontinuation**					**0.018**
MR^4−4.5^	8^*^	633	1,336	0.45 (0.38–0.52)	
≥MR^5^	4^*^	91	265	0.33 (0.26–0.41)	
TKI-WS					0.849
<27.3%	3	150	336	0.42 (0.31–0.53)	
≥27.3%	3	452	974	0.49 (0.26–0.54)	

* *before entering the TFR phase, the JALSG-STIM213 and STAT2 trials divided patients with deep molecular response into two subgroups (UMRD and MR4–4.5) and reported the number of relapses in each subgroup. Therefore, when studying the effect of depth of molecular response on TFR, the two studies were divided into two parts*.

#### Duration of DMR Before Entering the TFR Phase (Figure 3B, Table 3)

Median duration of DMR before entering the TFR phase was 32.8 months. The estimated loss of MMR was 35% (95%CI: 30–39) at 24 months in five (13, 14, 18, 22, 24) studies in which the criterion for imatinib discontinuation was ≥32.8 months of DMR. In the remaining four studies (15, 17, 21, 23) with duration of DMR <32.8 months, the estimated loss of MMR was 47% (95%CI: 42–52).

#### Depth of Molecular Response Before Entering the TFR Phase (Figure 3C, Table 3)

The estimated loss of MMR was 33% (95%CI: 26–41) at 24 months in four trials (13, 14, 22, 24), in which patients who achieved and sustained at least MR^5^ for several years were allowed to enter the TFR phase. In the remaining eight trials (10, 13, 15, 17, 18, 21, 23, 24), with patients sustaining <MR^4.5^ before discontinuation of TKIs, the estimated loss of MMR was 45% (95%CI: 38–52).

In addition to the above-mentioned findings, we found no significant difference in loss of MMR in subgroup analyses of age, sex ratio, Sokal score, type of first-line TKI therapy, treatment history, TKI-WS or total duration of TKI treatment before entering the TFR phase (Table 3). In addition, on sensitivity analysis by removing the low-quality study, the statistical significance of the 10 factors did not change.

## Discussion

This meta-analysis of observational studies of CML patients with stable DMR to TKIs focused on efficacy and safety of TKI discontinuation but also exploring the factors contributing to successful TFR. Estimated loss of MMR was 16, 34, 39, and 41% at 3, 6, 12, and 24 months, respectively, for patients who discontinued TKI treatment. Of these, 39, 82, and 95% of molecular losses occurred within the first 3, 6, and 12 months, respectively. Importantly, TFR had no negative impact on clinical outcomes because almost all patients (98%) who failed to remain TFR were sensitive to retreatment with the same TKI and regained MMR rapidly; no CML-related deaths occurred; and only one patient showed progression to blast crisis. In addition, our subgroup analyses suggested better TFR results associated with interferon therapy, depth of molecular response and duration of DMR.

Several limitations in the present study merit consideration. The major limitation concerns the discrepancy of study designs such as TKI type, accuracy and sensitivity of RT-qPCR assay, interferon therapy, and depth or duration of DMR before TKI discontinuation. Severe heterogeneity can result when pooling data with study design variations, particularly the definition of molecular recurrence, because it has a direct impact on the success rate of TKI discontinuation. The consensus on the definition of recurrence was originally derived from the A-STIM study, which confirmed that MMR loss is a more appropriate trigger for resuming TKI therapy; both NCCN and recently conducted TFR studies that adopted MMR loss to define recurrence. Therefore, in the literature screening, we specifically excluded studies with a stricter definition of recurrence such as the STIM and TWISTER studies. In addition, despite design differences, the observational studies we included had high methodological quality. Our results were not affected after a sensitivity analysis to exclude the low-quality study. Furthermore, subgroup analyses to explore prognostic factors contributed to the interpretation of heterogeneity. Another limitation is the lack of long-term follow-up data, with most studies reporting only ≤ 2 years follow-up. Considering the occurrence of very late relapses, longer follow-up data is needed to determine the long-term TFR rate. Finally, the number of trials included in this meta-analysis was limited and resulted in too few trials in some subgroups during subgroup analysis.

Although depth of molecular response and duration of DMR are two different predictors, we feel that the nature of the two predictors acting on TFR is similar; namely, lower level of BCR-ABL1 transcripts before TKI discontinuation is favorable for TFR. The reasons for this conjecture are based on the following: first, DMR is recognized as a prerequisite for attempting TFR, indicating that low level of BCR-ABL1 transcripts is the cornerstone for successful TFR; second, both ISAV (21) and KID (14) studies used digital PCR with better quantitative accuracy than conventional RT-qPCR and showed undetectable minimal residual disease (MRD) as better for TFR; third, the transcript level of BCR-ABL1 gradually decreases with TKI treatment, and, depending on treatment duration, even after transcripts become undetectable, residual leukemia may be further reduced with continued treatment (25). In summary, stable low transcript level of BCR-ABL1 does not exclude successful TFR but likely increases the probability of molecular relapse. Access to high-precision techniques such as digital PCR that may able to detect MRD and its dynamics during the TFR phase remain the key to elucidating the association between BCR-ABL1 transcript level and TFR outcomes. Another potential explanation for the role of low transcript level of BCR-ABL1 in promoting TFR might be the immune system. Previous studies have shown an association of increased proportion of immune effectors with successful TFR, which may maintain TFR by inhibiting CML cells (16, 26). Similarly, our study also suggests the positive effect of interferon treatment on TFR probability.

Recently, Bocchia et al. (27) reported no correlation between transcript level of BCR-ABL 1 and number of circulating CD26^+^ leukemic stem cells (LSCs) in patients on or off TKI treatment. This conclusion is beneficial for achieving TFR, because LSCs do not depend on BCR-ABL1 for their survival, thereby resulting in their inability to be cleared by TKIs. Also, Bocchia et al. (27) detected circulating CD26^+^ LSCs in 66% of CML patients in the prolonged and stable TFR phase, which suggests that eradication of these cells is not necessary for maintaining TFR in most patients. However, as compared with the initial diagnosis, the number of circulating CD26^+^ LSCs is decreased to low or undetectable levels during TKI treatment or TFR (27, 28). A possible LSC threshold for achieving and maintaining TFR still requires investigation.

We found no difference in TFR at 6, 12, and 24 months between patients receiving imatinib and second-generation TKIs. Of note, previous studies demonstrated that second-generation TKIs could induce a relatively faster and higher DMR as compared with imatinib (29–31). Moreover, in patients with failure of imatinib treatment for a prolonged time, a deeper molecular response can be obtained upon salvage with nilotinib (32). Thus, a reasonable strategy is using a more potent TKI to increase the number of candidates for discontinuing treatment, thereby potentially increasing the number of patients with successful TKI discontinuation, despite a need to weigh the potential serious toxicity of second-generation drugs (29). Similarly, previous studies identified several factors associated with higher DMR such as older age and interferon therapy (33–36), albeit with conflicting results. The availability of a prognostic factor for more accurate prediction of DMR would also be an important step in decisions for TKI discontinuation.

The assessment of BCR-ABL1 transcripts is critical in safety for patients attempting TKI discontinuation. However, to date, the standard molecular monitoring strategy after TKI discontinuation has not been established. Our findings show that 82 and 95% of molecular relapse occurred within the first 6 and 12 months, respectively. To ensure timely retreatment, we recommend molecular monitoring of BCR-ABL1 transcripts monthly during the first 6 months, then every 3 months until 12 months, then every 6 months until 24 months. Of note, very late relapses were rarely seen among patients with discontinued treatment but did occur occasionally even beyond the cutoff date (10, 13, 21). Thus, long-term follow-up with a 9-month monitoring interval seems reasonable; the specific schedule of molecular monitoring may rely on the individual patient level of fluctuating transcripts. In addition, patients with an increase in BCR-ABL1 level with a tendency for continued increase deserve more attention (even if MMR remains) because the transition from DMR to MMR loss may be sudden (37).

Misunderstandings about safety surrounding TFR are common in CML populations. A fear for patients considering discontinuation is that CML may relapse or even worsen after stopping TKI treatment and may subsequently not remain sensitive to TKI resumption (38). Our meta-analysis demonstrates that temporary TKI discontinuation is safe, because 98% of patients showing relapse regained MMR at the end of follow-up. Furthermore, no CML-related deaths were found in TFR studies, and only one patient showed progression to blast crisis. In addition, although the rate of AEs did not change much within the first year of TKI discontinuation, most drug-related AEs decreased over time after long-term follow-up (24).

However, about 27% of the patients who remained in the TFR phase showed newly developed or worsened TKI-WS consisting of musculoskeletal pain or pruritus within several weeks after stopping treatment. Many of these symptoms can last for months but, fortunately, are usually reversible, which was specifically confirmed in the ENESTfreedom study, in which the frequency of TKI-WS decreased from 34% during the first 48 weeks of TFR to 9% during the second 48 weeks (15). Indeed, except for a small number of patients with severe TKI-WS who require steroids for symptomatic treatment, most patients recovered with or without drug support.

## Conclusions

In ~59% of patients with sufficient TKI response, TFR is clinically feasible as an extension of an approach to optimize management of CML. In the remaining 41% of patients with molecular relapse, discontinuation of TKI treatment had no negative impact on clinical outcomes. To expand the opportunities for remaining treatment-free, interferon therapy, depth of molecular response and duration of DMR might be important considerations. Given the high heterogeneity among studies, more clinical trials as well as biological investigations are needed to determine the preconditions for achieving TFR.

## Author Contributions

KC, GF, and WY contributed to the conception and design this study. KC and PX developed the methodology. KC and TD analyzed and interpreted the data. KC and TD wrote the manuscript and approved the final submission of the study. All authors read and approved the final manuscript.

### Conflict of Interest Statement

The authors declare that the research was conducted in the absence of any commercial or financial relationships that could be construed as a potential conflict of interest.
