# Supplementary material for: Discontinuation of Tyrosine Kinase Inhibitors in Chronic Myeloid Leukemia With Losing Major Molecular Response as a Definition for Molecular Relapse: A Systematic Review and Meta-Analysis
Source: Front Oncol. 2019 May 14;9:372. doi: 10.3389/fonc.2019.00372 (PMC6527744; doi:10.3389/fonc.2019.00372)
Supplement: Supplementary file 1 [file Table_1.DOC]

Table 1. Risk of bias

| **Study** | Study population clearly defined | Cohort representative for the CML-CP patients with sufficient TKI response under TFR conditions | Definition of molecular response according to the IS | Demonstration that outcome of interest was not present at start of study | Consecu  -tive patients included | Assess  -ment of outcome | Follow up long enough for outcome to occur (≥2 years) | Adequacy of follow up of cohorts | Prospec-tive studies |
| --- | --- | --- | --- | --- | --- | --- | --- | --- | --- |
| Takahashi,N.(2012) | **-** | **-** | ★ | **-** | **-** | ★ | ★ | ★ | **-** |
| Rousselot,P.(2013) | ★ | ★ | ★ | ★ | ★ | ★ | ★ | ★ | ★ |
| Mori,S.(2015) | ★ | **-** | **-** | ★ | ★ | ★ | ★ | ★ | ★ |
| Lee,S.E.(2016) | ★ | ★ | ★ | ★ | ★ | ★ | ★ | ★ | ★ |
| Ross,D.M.(2018) | ★ | ★ | ★ | ★ | ★ | ★ | ★ | ★ | ★ |
| Rea,D.(2017) | ★ | **-** | ★ | ★ | ★ | ★ | ★ | ★ | ★ |
| Takahashi,N.(2017) | ★ | ★ | ★ | ★ | ★ | ★ | ★ | ★ | ★ |
| Takahashi,N.(2018) | ★ | ★ | ★ | ★ | ★ | ★ | ★ | ★ | ★ |
| Susanne (2018) | ★ | **-** | ★ | ★ | ★ | ★ | ★ | ★ | ★ |
| Mahon,F.X.(2018) | ★ | ★ | ★ | ★ | ★ | ★ | ★ | ★ | ★ |

Abbreviations: CML: chronic myeloid leukemia; CP: chronic phrase; IS: international scale; ★: yes; **-:** no
