# Supplementary figures and images for: Discontinuation of Tyrosine Kinase Inhibitors in Chronic Myeloid Leukemia With Losing Major Molecular Response as a Definition for Molecular Relapse: A Systematic Review and Meta-Analysis
Source: Front Oncol. 2019 May 14;9:372. doi: 10.3389/fonc.2019.00372 (PMC6527744; doi:10.3389/fonc.2019.00372)

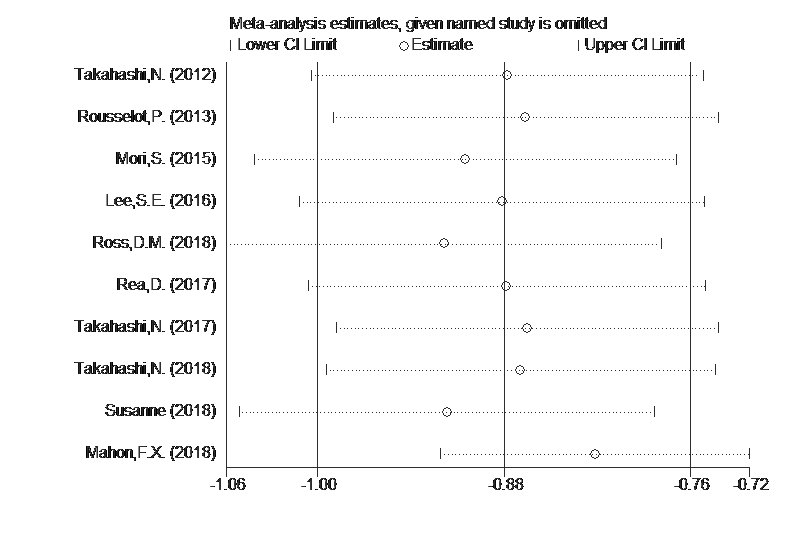

Supplement: Supplementary file 2 [file Image_1.TIF]
